# Public Health and Nursing: A Natural Partnership

**DOI:** 10.3390/ijerph6112843

**Published:** 2009-11-16

**Authors:** Christine Savage, Joan Kub

**Affiliations:** 1 University of Cincinnati College of Nursing, 3110 Vine St., Cincinnati, OH 45221, USA; 2 Johns Hopkins School of Nursing, Baltimore, MD 21205, USA; E-Mail: jkub@son.jhmi.edu

**Keywords:** public health, nursing, health care workforce

## Abstract

The health of individuals occurs within the context of their environment and the other individuals they interact with in the communities they live in, work in and visit. Promoting the health of the public requires multiple strategies aimed at improving the environment, the health knowledge of groups and individuals, maintaining adequate food and water, and reducing the spread of disease. Many disciplines are needed to meet these goals, but the largest segment of the professional health work force required to meet these needs is nursing. Historically, nursing leaders in public health such as Florence Nightingale and Lillian Wald made significant inroads related to serious health issues because they were nurses. Today across the globe, nurses provide the key components of public health interventions including well baby care, health education, screening and immunization clinics, disaster management and emergency preparedness. With the growing nursing shortage in acute care settings, the brain drain of nurses from certain areas of the world, the shrinking public dollars for preventive health care, the nursing workforce needed to continue to provide these essential health care services is threatened. It is essential to put the spot light on nursing’s role in public health with the hopes of attracting more public funds and more nurses to provide these essential services.

## 1. Introduction

The Institute of Medicine (IOM), in their report *The Future of the Public’s Health* defined public health as what society does collectively to assure the conditions for people to be healthy [1]. This definition builds on the classic definition of public health proposed by C.E. Winslow in 1920, which indicates that public health is the science of preventing disease, prolonging life and promoting health through organized community efforts [2]. Since Florence Nightingale, nursing has been an explicit part of the planning, evaluation and implementation of actions taken to meet the goals of public health. The continued efforts needed to improve the health of populations and communities require not only an appreciation of the role nurses have played in the past, but a clear explicit commitment from both the nursing profession and the field of public health science that nurses, the largest segment of the professional health care workforce are key to the success of meeting public health goals across the globe.

According to the World Health Organization (WHO), “In the 21st century, health is a shared responsibility, involving equitable access to essential care and collective defense against transnational threats.” [3]. Nurses share in this responsibility. In baccalaureate undergraduate nursing programs in the United States community/public health is viewed as essential content. Specialty programs in public health nursing exist at the master’s degree and doctoral degree level. That collective defense against transnational threats requires that nurses develop skills in public health science so that practice related to prevention is informed and tied to a clear knowledge base. A clear example of this is the outbreak of H1N1 Swine Flu virus. Though individual risks such as age and immune status contribute to the severity of an infectious disease once acquired, prevention is determined by access to adequate sanitation, herd immunity, living environments and in the case of H1N1 and other infectious diseases, vaccines. Professional nurses are being called to deliver the vaccines against this global threat and most importantly to provide the needed education to the public to prevent the spread of the disease. School nurses in fact were essential in the detection of the virus, helping to abate the initial spread of the disease.

## 2. Historical Perspectives of Public Health and Nursing

The intertwined history of nursing and public health can be traced back to the modern founder of the nursing profession, Florence Nightingale, a recognized early pioneer in epidemiology. Prevention and public health science. She developed a system of training for nurses that changed nursing from an ill defined job with no consistent method of entry to a true profession with a clear educational foundation required for entry into the profession. She also advanced the science of public health through her work on sanitation, surveillance and her dedication to social reform [4]. She shed light on the dismal conditions related to the health care available to the sick poor as well as their living and working conditions. During the Crimean War her statistical records on the living conditions of the soldiers demonstrated the relationship between sanitation and the presence of disease. Her ground breaking work changed the care of soldiers in the field and translated into a new understanding of the need to implement improved sanitation practices in all health care settings.

Nursing’s focus on prevention continued beyond the efforts of Florence Nightingale. A recent volume of the *British Journal of Nursing* provided an overview of the articles included in a 1909 issue of the journal. The main article from that 1909 issue highlighted in this report presented the role of the nurse in preventing blindness in newborns related to opthalmitis secondary to gonorrhea or chlamydia infection at birth. The recommended approach to prevention of blindness in an infected infant was to have continuous cleansing of the eye by a trained nurse. This issue included another article related to prevention of diphtheria and scarlet fever. The authors of the article on diphtheria suggested that nurses could be instrumental in stopping an epidemic. It is clear that 100 years ago nurses were seen as primary players in prevention of infectious diseases [5].

The role of the nurse in prevention was not only emphasized in Britain but in the United States as well. In the early part of the 20^th^ century in the United States, nursing and public health joined forces. Lillian Wald provided leadership to nursing when she founded the Henry Street Settlement located in New York City. The work of the Henry Street Nurses was to directly address the multiple causes of illness and also to create demonstration projects for purpose of preventing illness. Wald believed that treatment of illness by nurses required that they also treat social and economic problems that contributed to the illness [6]. The nurses visited families in the New York tenements with an ill family member and used the opportunity to educate the families on principles of basic sanitation and nutrition. The work of these nurses and others like them contributed to the decline of infectious diseases such as tuberculosis long before modern medicine was available. Wald is remembered for not only coining the term “public health nurse” but also providing a paradigm of holistic practice emphasizing multiple determinants of health and the importance of the environment in influencing health outcomes.

### 2.1. The Role of Public Health Nursing in Global Public Health Today

Public health nurses are key members of the interdisciplinary team needed to meet the demands of public health today. A starting point for understanding the role of the nurse in public health is to examine the role of nurses who have the title of public or community health nurse and to look at the key activities they engage in related to promoting the health of populations.

Numerous attempts have been made globally to describe the multiple roles nurses take on related to promoting the health of populations. Two models recently published, one related to Scotland and one from Japan, demonstrated that nursing services that positively impact the health of communities extends across multiple levels of care including nurses visiting in the home, providing care in the schools, district nursing and family health nursing [7,8]. Bennett, Perry and Lawrence made a strong argument that nurses working in primary care significantly contribute to the goals of promoting and protecting health [9]. In the United States, public health nursing is the largest professional group (10%) of public health workers in the U.S working in a wide range of settings including health departments [10].

Today across the globe, nurses provide key components of public health interventions including but not limited to well baby care, health education, screening and immunization clinics, disaster management and emergency preparedness. Not only are they needed due to the sheer number of nurses prepared at both the generalist and specialty level in public health, but they bring a unique set of skills needed to plan, implement and evaluate public health interventions. They have knowledge of disease and wellness based on both their education and experience that includes the understanding the clinical implications of public health interventions, as well as the etiology and natural history of disease. Nursing practice is grounded in a holistic approach and provides an ideal bridge between individual care and the care of populations and communities. Nurses are educated to see the whole person and to see individuals within the context of their communities and culture. Without nurses, key public health functions would not occur at the level they are now provided. An example is the 2009 H1N1 pandemic. Nurses are being mobilized to not only provide the immunizations, but have an active role in the planning of mass immunization and implementation of prevention strategies. They not only safely administer the vaccines, but they provide appropriate health education to recipients of the vaccine and their families. As school nurses they implement environmental and behavioral changes to reduce spread of the virus and help to develop outreach programs to help immunize those at high risk. They are front line workers in public health because their nursing skills and knowledge enrich the public health team.

An argument can also be made that nurses in all settings are engaged in the principles of promoting and protecting the health of individuals. Most nurses engage in informal health promotion through various methods during each encounter with an individual and in their day to day care of multiple individuals. This includes health education, where they deliver their care, such as use of precautions to prevent the spread of infectious diseases and input into the policies of the organizations that employ them.

On a more formal basis, promoting safe environments even within an acute setting can be seen as a dimension of public health. During an outbreak, nurses play a key role in all stages of the outbreak including surveillance, case finding and analysis of the data. Most hospitals employ a nurse to run their infectious disease program. This nurse partners with all the nurses in the organization and with public health departments to address both hospital acquired and community acquired infections. In the event of an epidemic or pandemic, public health departments and health care organizations call on nurses to develop programs to address the need to protect the population through education and the administration of mass vaccinations. In other cases as seen in this particular issue, public health nurses are focusing on populations at risk needing interventions to address the prevention or amelioration of high risk conditions, whether it is chronic illness or mental health needs.

### 2.3. Challenges for the Future

Despite the long history of nursing’s role in public health and the fact that the largest subpopulation of public health workers at least in the United States are public health nurses, most nurses are employed by organizations that provide care to persons who are already ill and are often at the end stage of disease. The majority of nursing education at the undergraduate level focuses on the delivery of acute care rather than prevention. In the United States, reimbursement for prevention services is limited, reducing the incentive for nurses to become involved in preventive care. Work load in acute care settings can be high, leaving little time for health education with individuals and families during an episode of care.

We are at a crossroad as we think about the role and value of public health nursing. There is a definite need for an understanding of prevention by all nurses. There are also health needs that demand a population perspective in order to plan broad based interventions. The public health nurse draws on the skills of nursing but also the specialized knowledge of public health. The skills of the public health nurses for the future demand knowledge of evidenced based interventions, new paradigms to address new global challenges. Roles for the future will demand the ability to provide and plan public health services within multiple settings and to conduct research and evaluate programs.

## 3. Conclusions

The clear relationship between public health and nursing began in the 19^th^ century and continues today. Our founders provided us with paradigms and models that are exemplary. Public health nursing is truly a synthesis of nursing and public health. Nurses represent the largest segment of the professional health care workforce. Without nurses, much of the public health interventions essential to improving the health of individuals, families, and populations would not be possible on a large scale. Nurses who specialize in public health nursing provide the leadership to the nursing profession for addressing the core functions of public health, assessment, assurance, and policy development. Collectively, the nursing care provided to individuals, families and communities across the globe by nurses and public health nurses significantly contributes to the health of the communities they work in. This vital resource may be under recognized and underutilized in the implementation of formal programs to promote and protect the public’s health.

Both the nursing profession and the field of public health should champion a more explicit application of public health practices across all settings by nurses, If this resource were truly tapped, more rapid advances in meeting the global health goals of the WHO may be realized. With the growing nursing shortage in acute care settings, and the shrinking public dollars for preventive health care, the nursing workforce needed to continue to provide these essential health care services is threatened.
